# Chromosome aberrations in a large series of spontaneous miscarriages in the German population and review of the literature

**DOI:** 10.1186/1755-8166-7-38

**Published:** 2014-06-05

**Authors:** Jutta Jenderny

**Affiliations:** 1 Humangenetik, Labor Lademannbogen, Lademannbogen 61-63, DE-22339 Hamburg, Germany

**Keywords:** Chromosome aberrations, Cytogenetic analysis, Cytogenetics, Karyotyping, Miscarriage, QF-PCR

## Abstract

**Background:**

In a review of the literature in 2000 the different cytogenetic aspects of spontaneous miscarriages were well documented. This review also included the spontaneous miscarriage results of one large German study published in 1990. However, to our knowledge there are no new data on spontaneous miscarriages in the German population. Therefore, the aim of the present retrospective large study was to find out the incidence and types of chromosome aberrations in an unselected series of spontaneous miscarriages in the German population, and whether our more recent results were different to data published previously. In case of culture failure we implemented a quantitative fluorescent polymerase chain reaction (QF-PCR) for chromosomes 13, 18, 21, X and Y.

**Results:**

In the present German retrospective study cytogenetic analysis (CA) was attempted on 534 spontaneous miscarriages between weeks 7 and 34 of gestation, being successful in 73% (390/534) of them. Two hundred and thirty-seven of the cases (61%, 237/390) were chromosomally abnormal. Trisomy was the most common chromosome aberration and accounted for 53% (125/237) of the aberrant karyotypes. A multiple aneuploidy was observed in 7% (17/237) of the aberrant karyotypes. Chromosomes 16, 22, 15 and 21 were found most frequently involved in aneuploidies. Fifty-four cases (23%, 54/237) with a polyploidy were found in the present study. Single unbalanced structural chromosome aberrations accounted for 4% (10/237) of the aberrant karyotypes. Eleven samples (5%, 11/237) displayed a variety of numerical and/or structural chromosome aberrations. One hundred and forty-four spontaneous miscarriages (27%, 144/534) failed to grow in culture. A total of 27 cases were analysed by QF-PCR for chromosomes 13, 18, 21, X and Y, being informative in all cases.

**Conclusion:**

In our German retrospective large study of spontaneous miscarriages, the incidence and types of chromosome aberrations by CA are within the reported range of other studies published previously before and after 2000. Therefore, we can conclude that cytogenetic aspects of spontaneous miscarriages have not changed over the years. Additionally 8 of 27 cases (30%) without cell growth showed a numerical chromosome aberration by QF-PCR. Therefore QF-PCR played an important role as a supplementary test when culture failure occurred.

## Background

Of all clinically recognized pregnancies, about 10%-16% end in early spontaneous miscarriages [1,2]. The overall prevalence of pregnancy losses is generally assumed to be much higher. Using an immunoradiometric assay to detect increased hCG levels near the expected time of implantation, 22% of pregnancies were ended before clinically detected [3].

Many factors can cause embryo losses, but it is well known from previous large studies (>500 cases) that up to 57% of spontaneous miscarriages result from chromosome aberrations [4-6]. Therefore cytogenetic analysis (CA) of the aborted sample is highly recommended and any result will facilitate counselling for affected couples [7]. In a review of the literature in 2000 the different cytogenetic aspects of spontaneous miscarriages by CA were well summarized in detail [8]. This review also included the CA results of a large German spontaneous miscarriage study published in 1990 [4]. However, new CA data on spontaneous miscarriages in the German population are rare. Therefore, the aim of the present retrospective large study was to find out the incidence and types of chromosome aberrations in an unselected series of spontaneous miscarriages in the German population, and whether our more recent CA results were different to data previously published.

CA of spontaneous miscarriages has a significant failure rate when the tissue sample (chorionic villi is the tissue of choice) is autolysed or otherwise not suitable for standard cell culture. Since 2000, quantitative fluorescent polymerase chain reaction (QF-PCR) analysis has been well established in our laboratory [9-11], and we have confirmed the clinical utility of QF-PCR on prenatal chorionic villi [10]. To improve the success rate of CA on spontaneous miscarriages in our laboratory, a supplemental diagnostic assay was therefore considered. We implanted QF-PCR to detect major clinically significant numerical chromosome aberrations in spontaneous miscarriages, and in case of cell growth failure, molecular analysis for chromosomes 13, 18, 21, X and Y was performed on demand.

## Methods

A total of 534 spontaneous miscarriage cases were collected between 2002 and 2013, most of them were referred to our laboratory without any clinical data. All the samples consisted of chorionic villi obtained after surgical evacuation between weeks 7 and 34 of gestation age. Uncertain materials were histologically examined by the pathologists in our laboratory. Selected chorionic villi were examined to exclude maternal deciduas remains. The carefully dissected chorionic villi samples were cultured and karyotyped according to a standard procedure. A nearly direct-preparation of chorionic villi was not carried out, because this method is too labour-intensive in a routine diagnostic laboratory. At least 11 G-banding metaphases were analysed. The G-banding quality was between 400 to 500 bphs. The aberrations and karyotypes were classified according to the International System for Human Cytogenetic Nomenclature 2013 (ISCN 2013) [12]. A mosaicism was defined according to the ISCN 2013 definition of a clone: loss of a chromosome must be detected in at least three cells, gain of a chromosome and/or a structural chromosome aberration must be present in at least two metaphases. In case of cell growth failure QF-PCR was performed on demand. Between 2002 and 2011 genomic DNA was extracted from the cells of the tissue culture without cell growth using a QIAamp blood kit (Qiagen, Germany). From 2012 onwards genomic DNA was extracted from a small piece of the chorionic villi within the first 7 days after sample collection using a QIAamp blood kit (Qiagen, Germany). Extracted DNA was stored frozen at minus 20°C until the decision of the patient whether a QF-PCR investigation should be carried out. For the detection of a clinically significant numerical chromosome aberration the conditions of the QF-PCRs with 13 selected specific markers were described previously [9]. From 2012 onwards, we used the Devyser Compact v3 kit with 26 selected specific markers according to the instructions of the company (Devyser AB, Stockholm, Sweden).

## Ethical approval and consent

These studies were performed on anonymized samples received in our laboratory and thus were exempted from the requirement for consent by an opinion for the Western Institutional Review Board.

## Results

CA was exclusively done from carefully dissected chorionic villi. CA from unidentified tissue and from induced abortions sent to the laboratory for confirmation of abnormal results obtained from CVS or amniocenteses were not included in the study. CA was attempted on 534 spontaneous miscarriages, being successful in 73% (390/534) of the samples. Of them, 42% (164/390) of the cases showed a male karyotype and 58% (226/390) of the cases showed a female karyotype. A normal male karyotype was observed in 74 of the cases and a normal female karyotype in 79 of the cases, resulting in a male-to-female sex ratio of 0.94. Two hundred and thirty-seven (61%, 237/390) of the cases were chromosomally abnormal. One hundred and forty-four spontaneous miscarriages (27%, 144/534) failed to grow in culture. Of them, a total of 27 cases were analysed by QF-PCR. QF-PCR analysis for chromosomes 13, 18, 21, X and Y was informative in all cases. An uninformative QF-PCR pattern for a single chromosome was not observed.

Table 1 shows the incidence and types of chromosome aberrations by CA in our retrospective large German study and reviewed other studies published previously. Table 2 lists the different CA and QF-PCR results obtained in the present study.

**Table 1:** Incidence and types of chromosome aberrations in spontaneous miscarriages by cytogenetic analysis (CA): new data from the German population and previous results from other studies published since 2000

**Study**	**Number of cases n**	**No results ** **n (%)**	**Normal karyotypes n (%)**	**Aberrant karyotypes n (%)**	**Monosomy X, Autosomal Monosomy, Mosaic aneuploidy n (%)**	**Trisomy** ** (pure) n (%)**	Triploidy*^1^, Tetraploidy*^1^, Polyploidy*^1 ^**n (%)**	Structural chromosome aberrations*^1 ^**n (%)**	Multiple aneuploidies*^1 ^**n (%)**	Others*^1 ^**n (%)**
Present study	534	144(27)	153(39)	237(61)	16(7), 1(<1), 17(7)	111(47)	29(12), 25(10), 54(23)	10(4)*^2^	17(7)*^3^	11(5)
Gao and coworkers [13]	100	14(14)	37(43)	49(57)	2(4), 1(2), 0	40(82)	3(6), 2(4), 5(10)	1(2)	0	0
Lathi and coworkers [14]	30	0	10(33)	20(67)	0, 0, 0	14(70)	1(5), 1(5), 2(10)	2(10)	2(10)	0
Shearer and coworkers*^3^[6]	4,189	828(20)	1,627(48)	1,734(52) 1,836^*4^	186(10)*^4^, 13(1)*^4^, 74(4)*^4^	1,074(58)*^4^	223(12)*^4,5^, 55(3)*^4,5^, 278(15)*^4,5^	135(7)*^4,6^	57(3)*^4^	19(1)*^4^
Menten and coworkers [15]	100	28(28)	55(76)	17(24)	2(12), 0, 0	10(59)	3(18), 0, 3(18)	2(12)	0	0
Dória and coworkers [16]	232	59(25)	107(62)	66(38)	5(8), 1(1), 3(5)	33(50)	6(9), 6(9), 12(18)	4(6)	7(11)	1(1)
Zhang and coworkers [17]	115	23(20)	37(40)	55(60) 53^*7^	5(9)^*7^, 0, 2(4)^*7^	36(68)^*7^	3(6)^*7^, 3(6)^*7^, 6(11)^*7^	2(4)^*7^	2(4)^*7^	0
Robberecht and coworkers [18]	103	26(25)	55(71)	22(29)	3(14), 0, 3(14)	7(32)	4(18), 0, 4(18)	3(14)	1(4)	1(4)
Diego-Alvarez and coworkers [19]	178	76(43)	62(61)	40(39)	6(15), 0, 0	24(60)	3(7), 2(5), 5(12)	1(2)	4(10)	0
Bruno and coworkers [20]	78	11(14)	38(57)	29(43)	2(7), 0, 1(3)	18(62)	1(3), 0, 1(3)	5(17)	1(3)	1(3)
Hu and coworkers [21]	38	7(18)	15(48)	16(52)	2(12), 0, 0	12(75)	2(12), 0, 2(12)	0	0	0
Menasha and coworkers [5]	2,180	260*^8^(12)	821(43)	1,099(57)	96(9), 13(1), 4(1)	721(66)	116(11)*^9^, 18(2)*^9^, 152(14)*^10^	46(4)	67(6)*^11^	0
Sullivan and coworkers [22]	150	17(11)	77(58)	56(42) 55*^12^	5(9)*^12^, 0, 0	35(64)*^12^	Not reported, Not reported, 12(22)*^12^	3(5)*^12^	0	0
Schaeffer and coworkers [23]	41	0	25(61)	16(39)	1(6), 0, 0	14(87)	0, 0, 0	1(6)	0	0
Jobanputra and coworkers [24]	57	5(9)	22(42)	30(58)	2(7), 1(3), 0	17(57)	4(13), 2(7), 6(20)	0	2(7)	2(6)
Tabet and coworkers*^13^[25]	21	0	12(57)	9(43)	1(11)	6(67)	1(11), 0, 1(11)	1(11)	0	0
Lomax and coworkers [26]	301	48^*13^(16)	98(39)	155(61)	12(8), Not reported, Not reported	Aneuploidy 111(72)	Not reported, Not reported, 25(16)	7(4)	0	0
**Summary**	**8447**	**1,546(18)**	**3,251(47)**	**3,650(53) 3,749(54)**	346(9)*^14^, 30(1)*^14^, 104(3)*^14^	2,283(61)*^14^	399(11)*^14^, 114(3)*^14^, 568(15)*^14,5,10^	223(6)*^14^	160(4)*^14^	35(1)*^14^
Goddijn and Leschot (Review)*^15^	Not reported	Not always reported	2,377(51)	2,319(49)	308(13), Not reported, Not reported	1,216(52)	Not reported, Not reported 481(21)	132(6)	Not reported	182(8)*^16^
Variations from (%)		(1-39)		(38-77)	(2-90)	(33-76)	(8-31)	(2-8)		
Eiben and coworkers [4]	983	233(24)	370(49)	380(51)	40(11), Not reported, Not reported	229(60)	46(12), 32(8), 78(20)	18(5)	11(3)	4(1)

CA data before 2000 are listed at the end of the table (Goddijn and Leschot, Review [8], Eiben and coworkers [4]).

*^1^Mosaicism included.

*^2^Single structural chromosome aberrations, all of them were unbalanced, see Table 2.

*^3^Shearer and coworkers [6] analysed a total of 5,555 specimens. Unidentified tissue accounted for 25% (1,366/5,555) of their cases, chromosome analysis exclusively in chorionic villi/fetal tissue specimens was successful for 80% (3,361/4,189) of the cases and identified a chromosome aberration (single or more) in 52% (1,734/3,361) of the cases by CA.

*^4^The frequencies were calculated from 1,734 chorionic villi/fetal specimens and from 102 unidentified tissue samples with an aberrant karyotype (n = 1,836).

^*5^Samples included cases with a near-triploidy or near-tetraploidy.

*^6^Ninety-one of the 135 cases (67%) were unbalanced structural chromosome aberrations, 6 of the 135 cases were balanced Robertsonian translocations with a female karyotype and likely represented maternal tissue.

*^7^The data we have used, are based on 53 cases with a chromosome aberration (see Table 1 in the publication of Zhang and coworkers [17]).

*^8^Sixty-five of the cases were contaminated and 195 of the cases showed no cell growth.

^*9^Samples only included cases with a non-mosaic triploidy or tetraploidy.

^*10^Samples included cases with a non-mosaic and mosaic triploidy or tetraploidy and/or with a near/pseudopolyploid karyotype.

*^11^The data we have used, are based on the results from Table 2 in the publication of Menesha and coworkers [5]. The detailed analysis of their cases with multiple aneuploidy is shown in Appendix A and the exact number of multiple aneuploidies is higher (8%, 85/1,099).

*^12^The data we have used, are based on 55 cases with a chromosome aberration (see Table 2 in the publication of Sullivan and coworkers [22]).

*^13^ Fourty-eight samples could not be analysed by both CA and comparative genomic hybridization/flow cytometry.

*^14^The frequencies were calculated from 3,749 aberrant karyotypes.

*^15^Including the data of Eiben and coworkers [4].

*^16^Including double and triple trisomies, mosaicism, hydatidiform mola, autosomal monosomy and miscellaneous.

**Table 2:** Incidence and types of chromosome aberrations in spontaneous miscarriages by cytogenetic analysis (CA) and by quantitative fluorescent polymerase chain reaction (QF-PCR)

**Type of chromosome aberration by CA (and QF-PCR)**	**Number of patients**
**Monosomy (n = 21)**	
45,X	16
**Monosomy X (QF-PCR)**	**1**
mos 45,X/46,XX	2
45,XX,-21	1
mos 45,XX,-21/46,XX	1
**Trisomy (n = 131)**	
47,XX,+2	2
mos 47,XY,+2/46,XY	1
mos 47,XX,+3/46,XX	1
47,XX,+4	3
mos 47,XX,+4/46,XX	1
mos 47,XX,+5/46,XX	1
47,XY,+6	1
47,XY,+7	1
47,XX or 47,XY,+8	5
47,XX or 47,XY,+9	5
mos 47,XX,+9/46,XX	2
47,XX or 47,XY,+10	4
47,XX or 47,XY,+13	6
**Trisomy 13 (QF-PCR)**	**2**
mos 47,XX,+13/46,XX	1
47,XX or 47,XY,+14	5
mos 47,XX,+14/46,XX	1
47,XX or 47,XY,+15	17
mos 47,XX,+15/46,XX	2
47,XX or 47,XY,+16	22
mos 47,XX,+16/46,XX	3
47,XX or 47,XY,+18	8
**Trisomy 18 (QF-PCR)**	**2**
47,XX or 47,XY,+20	2
47,XX or 47,XY,+21	10
**Trisomy 21 (QF-PCR)**	**2**
47,XX or 47,XY,+22	20
mos 47,XX,+22/46,XX	1
**Triploidy (n = 30)**	
69,XXX or 69,XXY	29
**Triploidy (QF-PCR)**	1
**Tetraploidy (n = 25)**	
92,XXXX or 92,XXYY	5
mos 92,XXXX or 92,XXYY/46,XY	20
**Structural chromosome aberration (single) (n = 10)**	
46,X,i(Y)(q10)	1
mos 46,X,i(Y)(p10)/46,XY	1
46,XY,der(1)t(1;15)(p36.1;q22.3)mat	1
mos 46,XX,add(4)(q?31)/46,XX	1
46,XY,add(5)(p15.3)	1
46,XX,add(6)(q21)	1
mos 47,XY,+i(12)(p10)/46,XY	1
46,XX,add(14)(p11.2)	1
mos 46,XX,i(20)(q10)/46,XX	1
mos 47,XY,+mar/46,XY	1
**Two chromosome aberrations and more (n = 28)**	
Multiple aneuploidies (n = 17)	
46,X,+21	1
48,XXY,+22	1
48,XX,+2,+22	1
48,XX,+8,+18	1
48,XY,+9,+15	1
48,XY,+12,+20	1
48,XY,+13,+21	1
48,XY,+16,+21	1
48,XX,+16,+22	1
49,XY,+12,+16,+21	1
48,XX,+20,+21	1
68,XXY,-21	1
68,XXX,-22	1
94,XXXY,+7,+7	1
94,XXYY,+17,+17	1
94,XXYY,+20,+20	1
mos 95,XXYY,+2,+21,+21/94,XXYY,+21,+21	1
Structural and/or numerical chromosome aberrations (n = 11)	
mos 45,X/47,XX,+mar	1
46,XX,der(2)t(2;3)(q37;p21),der(3)t(3;9)(p21;q13)	1
mos 47,XX,+4/47,XX,der(4)t(4;?)(q12;?)	1
47,XY,t(5;5)(p10;p10),+5	1
mos 46,XY,der(5)t(5;14)(q23;q11.2),+5,-4/45,XY,der(13;14)(p10;q10)/46,XY	1
mos 46,XX,del(8)(p10)/46,XX,i(8)(q10)	1
47,XY,t(9;13)(p22;q14.1),+13	1
46,XX,der(14;15)(q10;q10),+14	1
47,XX,der(15)t(3;15)(p10;q10),+21	1
91,XX,-3,+5,-6,t(8;19)(q22;q13.1)	1
mos 94,XXXX,+mar1x2/47,XX,+mar1/46,XX	1
**Chromosome aberrations by CA**	**237/390, (61%)**
**Chromosome aberrations by QF-PCR**	**8**
**Chromosome aberrations by CA and QF-PCR**	**245/417, (59%)**

### Trisomy

#### CA (n = 125)

Trisomy was the most common chromosome aberration and accounted for 53% (125/237) of the aberrant karyotypes. Of them, 89% (111/125) of the cases were pure trisomic, 11% (14/125) of the cases showed mosaicism. The most frequent type was trisomy 16 (25 cases), followed by trisomy 22 (21 cases), 15 (19 cases) and 21 (10 cases).

QF-PCR detected a trisomy in 6 additional miscarriages with *in vitro* culture failure.

### Monosomy

#### CA (n = 20)

Monosomy accounted for 8% of the aberrant karyotypes (20/237). Monosomy X was found in 90% (18/20) of the monosomies. Of them, 11% (2/18) showed gonosomal mosaicism 45,X/46,XX. Monosomy 21 accounted for 10% (2/20) of the monosomies. Of them, one case showed a monosomy 21 mosaicism.

QF-PCR detected a monosomy X in one miscarriage with *in vitro* culture failure.

### Triploidy and Tetraploidy

#### CA (Triploidy n = 29, Tetraploidy n = 25)

Triploidy accounted for 12% (29/237) of the aberrant karyotypes. Mosaicism was not observed. Tetraploidy accounted for 10% (25/237) of the aberrant karyotypes. Of them, 80% (20/25) showed a tetraploidy mosaicism.

QF-PCR detected a triploidy in one additional miscarriage with *in vitro* culture failure.

### Structural chromosome aberrations (single) (n = 10)

Single structural chromosome aberrations accounted for 4% (10/237) of the aberrant karyotypes, all of them showed an unbalanced chromosome aberration. Fifty percent of the chromosomally unbalanced cases showed mosaicism.

### Multiple aneuploidies (n = 17)

Multiple aneuploidies accounted for 7% (17/237) of the aberrant karyotypes. Of them, 53% (9/17) of the cases carried a double trisomy and 6% (1/17) of the cases showed a triple trisomy. There was one case (6%, 1/17) of combined trisomy 21 and monosomy X. Six cases (35%, 6/17) showed a near-triploid or near-tetraploid karyotype. Gain or loss of chromosome 21 was found most frequently (7 cases), followed by chromosome 22 (4 cases) and chromosome 16 (3 cases).

### Others (n = 11)

These samples displayed a wide variety of numerical and/or structural chromosome aberrations and accounted for 5% (11/237) of the aberrant karyotypes. Nine cases showed double structural chromosome aberrations (derivative chromosome, translocation, deletion and isochromosome) or a single structural chromosome aberration together with one or two aneuploidies. A near-tetraploid karyotype was found in two further cases, one of them with the gain of chromosome 5 and the loss of chromosomes 3 and 6 together with a reciprocal translocation and the other with the gain of two copies of the same marker chromosome together with a 47,XX,+mar/46,XX mosaicism.

## Discussion

In the present study the overall male-to-female sex ratio among the normal karyotypes was 0.94 in chorionic villi. A consistent feature during the large German study of Eiben and coworkers [4] was the excess of females in chromosomally normal spontaneous miscarriages (male-to-female ratio of 0.71). Using a nearly direct-preparation CA method of chorionic villi after short culture of approximately 24 hours, their unbalanced rate cannot be explained by maternal contamination. The results of Eiben and coworkers [4] provide the evidence for a female-specific developmental disadvantage at early stage of embryonic development, probably because they analysed most of the spontaneous miscarriages between weeks 5 and 13 of gestation age. We, however, cultivated the chorionic villi for a longer time, after surgical evacuation between weeks 7 and 34 of gestation age. Without any clinical data, we could not separate our spontaneous miscarriage results between early or late gestation age. However, in a study approximately similarly designed to our investigation, Shearer and coworkers [6] observed a remarkably constant sex ratio of 1.0 both by culturing of chorionic villi and by FISH after direct cell preparation of the same tissue type. Based on these data we can suspect that our nearly balanced male-to-female sex ratio in pregnancies without a chromosome aberration indicated that the majority of analysed chorionic villi after careful dissection was not contaminated with maternal cells.

The results of the present retrospective large German study and of other foreign spontaneous miscarriage studies since 2000 reviewed and pooled in Table 1 give one of the most comprehensive indications of the incidence and types of chromosome aberrations by CA observed to date in spontaneous miscarriages. In our German spontaneous miscarriage group the frequency of 61% of cases with a chromosome aberration detected by CA is comparable with the results of both large foreign studies [5,6] after karyotyping 5,383 spontaneous miscarriages overall. They found a frequency of chromosome aberrations ranging from 52% to 57%. In both studies CA was exclusively done after tissue culturing and gestation age at the time of abortion was unknown, so that their data compare well with our results. In the single large German study in 1990 a low frequency of 51% of chromosome aberrations in 750 karyotyped spontaneous miscarriages was reported [4]. However, the proportions of chromosome aberrations among the different spontaneous miscarriage studies always varied extremely. The review in 2000 of Goddijn and Leschot [8] reported an average frequency of chromosome aberrations of 49% in 4,696 karyotyped cases, which is comparable with the pooled data in 2014 with an average frequency of chromosome aberrations of 54% in 7,000 karyotyped spontaneous miscarriages (Table 1).

At any time, autosomal pure and mosaic trisomies comprise the largest single class of chromosome aberrations in spontaneous miscarriages with trisomy 16 as the most common trisomy [5,6,8]. Also in our study, trisomy 16 was the most frequently represented, accounting for 20% of all trisomies, followed by trisomy 22 (17%), trisomy 15 (15%) and trisomy 21 (8%). Including the cases with multiple aneuploidies, we observed nearly each autosomal trisomy, with the exception of trisomy 1, trisomy 11, and trisomy 19. In the large foreign investigation of Shearer and coworkers [6], only trisomy 1 was missed in 1,836 cytogenetic aberrant spontaneous miscarriages and in a similar study of Menasha and coworkers [5] trisomy 1 and trisomy 19 were the least common, with only one case of each found in 1,099 aberrant karyotypes. The reason why certain types of chromosome aberrations are infrequent is unknown. However, chromosome 1 and chromosome 11 are gene-dense [27,28] and chromosome 19 has the highest gene density of all human chromosomes [29]. Therefore trisomy of these chromosomes may be responsible for very early pregnancy loss.

Pure or mosaic monosomy X was always the predominant gonosomal aberration in all spontaneous miscarriage studies before and after 2000. Autosomal monosomies in spontaneous miscarriages are usually rare. Of the pooled cytogenetic results of 4,696 spontaneous miscarriages only five autosomal monosomies (0.2%) were reported per 2,319 aberrant karyotypes before 2000 [8]. In our study, we only observed two cases with monosomy 21 (0.8%). Monosomy 21 was also the most common autosomal monosomy in both large foreign studies published recently [5,6]. Shearer and coworkers [6] additionally observed one case with monosomy 13 and one case with monosomy 15 in 1,836 aberrant karyotyped samples.

Triploidy is not uncommon in early pregnancies (1% - 3% of recognized conceptions), but about >99% are lost during the first or second trimester. Of all 16-week pregnancies, only 1 in 30,000 is estimated to be triploid [30]. Surprisingly, Wick and coworkers [31] detected four cases of second-trimester triploidy (all patients were at least 19 weeks’ gestation) which were diagnosed at a tertiary centre within a 1-year period, so that some of the triploid karyotypes survived longer. Generally a high rate of triploidy has been observed in spontaneous miscarriages by CA. The frequency of 12% in the present study directly corresponds to the frequencies of other past large reports [4-6].

The tetraploidy rate of 10% in the present study is higher than the average frequency of tetraploidy of 3% in pooled data from 7,000 cytogenetically analysed spontaneous miscarriages (Table 1). If we only calculate our results with pure tetraploidy, the frequency would be 2.1%, which is in the range of other recent large studies [5,6]. Therefore, in the present study a cultural artifact in cases with tetraploidy mosaicism cannot be entirely excluded. However, it should be mentioned that Eiben and coworkers [4] reported a frequency of 8% of cases with a tetraploidy in the German spontaneous miscarriage group after nearly direct-preparation of chorionic villi with a rate of pure tetraploidy of 2.3%, which is in concordance with our results. In addition, because also their mosaic cases were classified independently by chorionic villi histology as being chromosomally abnormal Eiben and coworkers [4] assumed that a significant number of early spontaneous miscarriages might result from a disordered placental development characterized by chorionic mosaicism with diploid and tetraploid cells detectable only by their nearly direct-preparation technique. Possibly this phenomenon is detectable even after prolonged cultivation of chorionic villi. This would explain the high rate of mosaic tetraploidy cases in the present study.

In our spontaneous miscarriage group 7% (17/237) of all aberrant karyotypes carried a multiple aneuploidy. It is of interest that 15 out of the 17 affected patients were from age 35 to age 48, implying a direct correlation between increased maternal age with increased risk of non-disjunction of chromosomes. The collection of 85 multiple aneuploid karyotypes in 1,099 aberrant spontaneous miscarriages (8%) in the investigation of Menasha and coworkers [5] is the largest study published to date. These karyotypes were also detected predominantly in older women. The average frequency of multiple aneuploidies calculated from a pooled 3,749 aberrrant karyotypes obtained from conventional tissue culture is somewhat lower (4%, Table 1) and similar to the frequency (3%) of the past large German study of Eiben and coworkers [4] using a nearly direct-preparation CA method of chorionic villi. This reflects once more the apparent similarity between both culture techniques of chorionic villi or other heterogeneous tissues cultured from an aborted foetus.

The frequency of structural chromosome aberrations in spontaneous miscarriages seems to be extremely constant over many years. The pooled average frequencies of structural chromosome aberrations before 2000 and from 2000 to 2014 were 6% [8, Table 1]. In the present study, non-mosaic cases with a structural chromosome aberration, which could be inherited, occurred in 5% (11/237) of the aberrant karyotypes (Table 2). This frequency is the range of the reported frequency of 4% of unbalanced chromosome aberrations due to a translocation (n = 15) in the large German study in 1990 [4].

Although CA of spontaneous miscarriages is highly recommended, CA studies entail certain problems such as culture failure (Table 1). To overcome this problem, alternative techniques complementary to CA have been used for genetic testing of miscarriage samples, including fluorescence in situ hybridization (FISH), multiplex ligation-dependent probe amplification (MLPA) and QF-PCR [32]. In the present study, one hundred and forty-four spontaneous miscarriages (27%) failed to grow *in vitro.* As a supplementary method, we used QF-PCR to improve our success rate in the detection of clinically relevant chromosome aberrations. A total of 27 samples with culture failure were analysed by molecular analysis on demand. QF-PCR for chromosomes 13, 18, 21, X and Y was informative in all these cases and detected a chromosome aberration in a further 8 spontaneous miscarriages.

## Conclusion

By generating an overview of the genetic aspects of spontaneous and recurrent abortions, Van den Berg and coworkers [32] reported that more chromosome aberrations were detected by CA compared to FISH or MLPA or QF-PCR. Newer techniques such as array-comparative genome hybridization (array-CGH) or CGH are useful in detecting deletions and duplications including submicroscopic imbalances in spontaneous abortions, but they are unable to detect the ploidy status and low level mosaicism [32,33]. By using these techniques independently, instead of CA, they unfortunately showed no added clinical value [32]. Therefore, CA is still the gold standard in the detection of chromosome aberrations in spontaneous miscarriages until further work is done before the absolute detection rate can be answered with newer techniques. In our German retrospective large study, the incidence and types of chromosome aberrations by CA are within the reported range of other studies published previously before and after 2000. Therefore, we can conclude that cytogenetic aspects of spontaneous miscarriages have not changed over the years. Thereby, in our laboratory, QF-PCR played an important role as a supplementary test when culture failure occurred and improved our success rate in the detection of clinically relevant chromosome aberrations.

## Competing interests

The author declares that she has no competing interests.
